# Anti-CCR7 therapy exerts a potent anti-tumor activity in a xenograft model of human mantle cell lymphoma

**DOI:** 10.1186/1756-8722-6-89

**Published:** 2013-12-04

**Authors:** Beatriz Somovilla-Crespo, Manuel Alfonso-Pérez, Carlos Cuesta-Mateos, Cristina Carballo-de Dios, Amada E Beltrán, Fernando Terrón, Juan J Pérez-Villar, Carlos Gamallo-Amat, Gema Pérez-Chacón, Elena Fernández-Ruiz, Juan M Zapata, Cecilia Muñoz-Calleja

**Affiliations:** 1 Instituto de Investigación Sanitaria Princesa, Department of Immunology, Hospital Universitario de La Princesa, C/Diego de León 62, Madrid 28006, Spain; 2 Pathology, Hospital Universitario de La Princesa, Madrid, Spain; 3 Immunological and Medicinal Products, S.L., Madrid, Spain; 4 Instituto de Investigaciones Biomédicas “Alberto Sols”, CSIC-UAM, Madrid, Spain

## Abstract

**Background:**

The chemokine receptor CCR7 mediates lymphoid dissemination of many cancers, including lymphomas and epithelial carcinomas, thus representing an attractive therapeutic target. Previous results have highlighted the potential of the anti-CCR7 monoclonal antibodies to inhibit migration in transwell assays. The present study aimed to evaluate the *in vivo* therapeutic efficacy of an anti-CCR7 antibody in a xenografted human mantle cell lymphoma model.

**Methods:**

NOD/SCID mice were either subcutaneously or intravenously inoculated with Granta-519 cells, a human cell line derived from a leukemic mantle cell lymphoma. The anti-CCR7 mAb treatment (3 × 200 μg) was started on day 2 or 7 to target lymphoma cells in either a peri-implantation or a post-implantation stage, respectively.

**Results:**

The anti-CCR7 therapy significantly delayed the tumor appearance and also reduced the volumes of tumors in the subcutaneous model. Moreover, an increased number of apoptotic tumor cells was detected in mice treated with the anti-CCR7 mAb compared to the untreated animals. In addition, significantly reduced number of Granta-519 cells migrated from subcutaneous tumors to distant lymphoid organs, such as bone marrow and spleen in the anti-CCR7 treated mice. In the intravenous models, the anti-CCR7 mAb drastically increased survival of the mice. Accordingly, dissemination and infiltration of tumor cells in lymphoid and non-lymphoid organs, including lungs and central nervous system, was almost abrogated.

**Conclusions:**

The anti-CCR7 mAb exerts a potent anti-tumor activity and might represent an interesting therapeutic alternative to conventional therapies.

## Background

The metastatic spread of cancers takes place when neoplastic cells leave the anatomic boundaries of the affected organ. Conversely, the dissemination of lymphomas does not always reflect the progression of the tumor, but recapitulates the so-called homing signature of normal lymphoid cells, which is characterized by a conserved pattern of migration and recirculation [1,2]. This particular tissue tropism explains the rapid dissemination of lymphomas and the different patterns of tissue infiltration of the lymphoproliferative disorders [1]. The targeted lymphoid organs, whose microenvironment provides proliferative and survival signals to the tumor cells, become authentic sanctuaries for lymphoid malignancies [3,4]. Thus, controlling the lymphoma dissemination represents one of the unresolved therapeutic challenges in this type of neoplasia [5,6].

Homing of normal lymphoid cells is a multistep process that requires chemotaxis, cell adhesion, and extravasation of lymphocytes across the vessel wall. This process is regulated by adhesion molecules and chemokine receptors on the surface of the lymphocytes, and their ligands expressed by the endothelial cells [7,8]. CC-chemokine receptor 7 (CCR7) is a well-characterized chemokine receptor that is expressed on naïve and central memory lymphocytes and mature dendritic cells and this allows these cells to respond to the ligands of CCR7, the homeostatic chemokines CC-chemokine ligand 21 (CCL21) and CCL19, produced in secondary lymphoid organs (SLO) [9]. CCR7 is required for the entry of normal T and B lymphocytes through the endothelium of high endothelial venules into the SLO, including lymph nodes and Peyer’s patches [10,11]. Consistent with their lymphoid origin, many leukemias and lymphomas express CCR7 [12-16]. Indeed, results from our laboratory have demonstrated that CCR7 plays a major role in the migration and nodular dissemination of certain lymphoproliferative syndromes including chronic lymphocytic leukemia (CLL) and mantle cell lymphoma (MCL) [12]. In addition, CCR7 also plays a significant role in the lymph node dissemination of those epithelial solid tumors that ectopically express this chemokine receptor [17]. Furthermore, CCR7 has been also implicated in acute T-cell leukemia infiltration of the central nervous system (CNS) [18].

Therefore, the blockage of CCR7-mediated migration might represent a new therapeutic approach for the treatment of certain lymphoproliferative disorders. In this regard, we previously demonstrated that anti-CCR7 antibodies and different chemical inhibitors of the signaling pathways activated by CCR7 efficiently blocked *in vitro* migration of primary CLL cells in response to the CCR7 ligands. Moreover, our results also showed that anti-CCR7 antibodies induced potent *in vitro* Fc-mediated complement-dependent cytotoxicity [19,20].

These *in vitro* findings have led us to investigate the *in vivo* efficacy of anti-CCR7 therapy. Among the different CCR7-expressing hematological tumors, we decided to study the benefits of an anti-CCR7 mAb on MCL due to the limited therapeutic options and an unmet need of alternative treatments for this hematologic disorder [21-23]. MCL is an aggressive B-cell malignancy that accounts for approximately 6% of all non-Hodgkin lymphoma (NHL) cases diagnosed every year. Current therapies include chemo-immunotherapy or high dose chemotherapy followed by autologous stem cell transplantation. Although conventional chemotherapy induces high-remission rates in previously untreated patients, relapse within a few years is common, contributing to a rather short median survival of 5–7 years [24,25]. In this regard, mAbs represent ideal alternative options for heavily pretreated patients with relapse and/or refractory MCL because their limited toxicity and the improvement of patient outcomes when combined with chemotherapy [26]. Interestingly, a recent meta-analysis indicated that the addition of rituximab to the conventional chemotherapy may increase the overall survival when compared with chemotherapy [27].

We hereby show that the treatment of MCL-xenografted mice with an anti-CCR7 mAb significantly increased the survival of the animals. The increased survival was due to both decreased infiltration of MCL cells into different tissues and to the induction of MCL cells cytotoxicity in the mice. In summary our results support that anti-CCR7 immunotherapy might be an option for the treatment of MCL and other CCR7+ lymphoproliferative disorders.

## Methods

### Cells and culture

Granta-519 human mantle cell lymphoma (MCL) cell line was purchased from the German Collection of Microorganisms and Cell Cultures (DSMZ) repository (Braunschweig, Germany). Cells were cultured at 0.5-2.0 × 10^6^ cell/ml in RPMI-1640 supplemented with 10% (v/v) fetal calf serum (FCS), 2 mM L-glutamine, 100 unit/ml penicillin and 0.1 mg/ml streptomycin. Experiments with human specimens were approved by the ethics committee of the Hospital de la Princesa. Human samples were obtained from healthy donors and from patients with different B-cell neoplasms after informed consent. Human peripheral blood (PB) and bone marrow (BM) aspirates were obtained by venipuncture and sternun puncture, respectively, and peripheral blood mononuclear cells (PBMC) were separated by ficoll density gradient centrifugation.

Murine splenocytes were obtained from NOD/SCID and NSG mice by splenectomy and separated by ficoll density gradient centrifugation.

### Reagents

Mouse anti-human CCR7 mAb (150503 clone, IgG2a isotype) was obtained from R&D Systems (Minneapolis, MN, USA) and was resuspended in sterile water.

Alemtuzumab was obtained from the department of pharmacy at our hospital.

For flow cytometric analysis, mouse anti-human CD19 mAb (fluorescein isothiocyanate; FITC), mouse anti-human CD20 mAb (Pacific Blue; PB), mouse anti-human CCR7 mAb (Phycoerythrin; PE) and the DNA dye 7-Actinomycin-D (7-AAD) were purchased from Becton Dickinson (BD) Biosciences (San José, CA, USA).

### CCR7 expression

CCR7 expression in Granta-519 cells was assessed by flow cytometry. Briefly, 1 × 10^6^ Granta-519 cells were washed twice with cold PBS, resuspended in 100 μl cold PBS, incubated with the PE-conjugated anti-human CCR7 mAb for 15 minutes and washed with PBS. An appropriate isotype control was included in the analysis.

For staining of primary samples, 100 μl whole PB or BM samples were incubated for 15 minutes at room temperature with PE-conjugated anti-human CCR7 mAb. This incubation was followed by the lysis of red blood cells by using ammonium chloride lysing solution (BD Biosciences) following the manufacturer’s instructions. Finally, leukocytes were resuspended on 500 μl cold PBS. Data acquisition and analysis were performed on a FACSCanto II flow cytometer using the DIVA software (BD Biosciences). In all experiments, a minimum of 5000 neoplastic B cells was acquired. Results are expressed as the percentage of CCR7 positive cells and mean fluorescence intensity (MFI) of CCR7 expression.

### Transwell assays

Chemotaxis of Granta-519 cells in response to CCL19 and to CCL21 was assayed in Transwell cell culture chambers (6.5 mm diameter, 10 μm thickness, 5 μm diameter pore size, Costar, Cambridge, MA). When required, cells were preincubated for 30 min with 2 μg/ml anti-CCR7 mAb. Briefly, 5 × 10^5^ Granta-519 cells, resuspended in 100 μl RPMI-1640 medium with 0.5% bovine serum albumin (BSA), were added to the upper compartment of the chamber, and chemokines were added to the lower well in 600 μl of the same medium at the optimal concentration (1 μg/ml for both CCL19 and CCL21). Migration was allowed to proceed for 4 h at 37°C in 5% CO2 atmosphere. Migrated cells were recovered from the lower chamber and counted by flow cytometry for 60 s after calibrating the flow rate with Trucount tubes (BD Biosciences). Events were compared with the number of cells counted in the initial suspension of cells to calculate the percentage of input (100 × number of cells migrated/number of cells counted in the initial suspension). Each experiment was performed in duplicate.

### Complement-dependent cytotoxicity (CDC)

1 × 10^5^ Granta-519 cells (50 μl) were seeded in a 96-well round-bottom plate together with 2 μg/ml of either purified anti-human CCR7 mAb or the corresponding isotype control (IC). After 30 min incubation at 37°C, the cells were centrifuged and washed. Then, baby rabbit complement (Serotec, Oxford, UK), diluted at the concentration indicated by the manufacturer (25%) in RPMI-1640 medium was added. After 1–2 h at 37°C, the cells were stained with fluorescein isothiocyanate (FITC)-conjugated anti-CD19 mAb and PB conjugated anti-CD20 mAb and with 7-AAD as a viability exclusion dye. The percentage of non-viable cells was measured and the percentage of lysis with heat-inactivated complement was used to calculate the specific lysis with the formula: Specific lysis (%) = 100 × (% dead cells with complement –% dead cells with inactivated complement) / (100–% dead cells with inactivated complement).

### Antibody-dependent cell-mediated cytotoxicity (ADCC)

ADCC assays were performed using Granta-519 cell line as target cells and either human PBMC or murine splenocytes as effectors cells. The target to effector ratio was 1:10 in both cases.

Granta-519 cell were washed and resuspended at 1 × 10^6^ cells/ml in PBS containing 5 μg/ml calcein-UV Cell Tracker (Invitrogen, OR, USA) and incubated at 37°C for 30 minutes. Cells were then washed twice and resuspended in RPMI-1640 supplemented with 10% FCS, in presence or absence of 100 μg/ml of either isotype control, anti-CCR7 mAb, or alemtuzumab for 30 minutes. Granta-519 cells were washed again and 1 × 10^5^ cells were plated with human PBMC or murine splenocytes. After 24 hours, the cells were stained with 7-AAD and analyzed by flow cytometry (FACSCanto II, BD Biosciences). The percentage of Granta-519 cells killed by antibody-mediated cytotoxicity was calculated substracting the percentage of dead cells in the presence of control isotype mAb.

### Mice

NOD/SCID and NSG female mice were housed in the animal facility of the Instituto de Investigaciones Biomédicas “Alberto Sols” and in the facilities of Vivotecnia, under standard sterile conditions in air-filtered containers, according to protocols approved by European directives and Spanish laws (European Directives 86/609/EEC/2003/65/EC Spanish Law RD 1201/2005). NSG mice were from Charles River (Barcelona, Spain). The *in vivo* experimental procedures were approved by the pertinent ethic committees and carried out in accordance with the guidelines of the European directives and Spanish laws.

Only those animals that met the inclusion criteria (20% of the mean body weight) were included in the study and distributed into the different experimental groups according to the body weight stratification method.

### In vivo anti-tumor activity of anti-human CCR7 mAb in NOD/SCID mice

To evaluate the anti-tumor efficacy of the anti-human CCR7 mAb, NOD/SCID mice were xenografted with the Granta-519 human MCL cell line. All mice used in the experiment were females and were 8 ± 1 weeks old.

We have used two inoculation *vias*: (1) cells were subcutaneously injected resulting in a localized tumor and (2) cells were intravenously injected resulting over time in a disseminated lymphoma.

The subcutaneous model was developed by inoculating a group of 5 mice with 5 × 10^6^ viable cells subcutaneously (in the right hind flank). The number of inoculated cells to establish the subcutaneous model was chosen on the basis of previous experiments to determine the number of Granta-519 cells required to develop palpable tumors in the mouse in around one week.

This subcutaneous model was used as an early-treatment model of the lymphoma and therefore the mice were intraperitoneally injected with 200 μg anti-human CCR7 mAb two days after inoculation of Granta-519 cells. This treatment was repeated on day 6 and 10. As a control group we inoculated a group of 5 mice with sterile PBS on days 2, 6 and 10.

The disseminated model involved inoculating mice intravenously (in the tail vein) with 0.5 × 10^6^ cells. The number of Granta-519 cells inoculated in the intravenous model was chosen on the basis of previous experiments done to establish the number of Granta-519 cells that resulted in the development of visible signs of disease in a period of around 40–60 days. This model was split into two branches, a “peri-implantation” model, defined as the period in which tumor cells are circulating and not yet located in the target organs, in which mice were treated 2 days after the xenograft, and a “post-implantation” model, in which surviving tumor cells are expected to have reached their target organs. In this model mice were treated 7 days after the xenograft. The peri-implantation model included a group of 5 mice treated with 200 μg anti-human CCR7 mAb intraperitoneally on days 2, 6 and 10. A control group of 5 mice were inoculated with PBS on the same days of 2, 6 and 10. The post-implantation model involved 3 groups of mice. A group of 5 mice were inoculated with 200 μg anti-human CCR7 mAb intraperitoneally on days 7, 11 and 15. A second group of 5 mice were a control group inoculated with 200 μg of an isotype control intraperitoneally on days 7, 11 and 15. And a third group also a control group that received sterile PBS inoculated on days 7, 11 and 15. Mice were weighted every three days and checked for any signs of pathologies, discomfort or mortality, according to the OCDE Humane Endpoints Guidance Document.

The length and width of the tumors in the subcutaneous model were measured with a caliper three times per week or when deemed necessary once the tumor lenght was ≥ 4 mm. The largest (D) and shortest (d) diameters of the tumor were measured every third day and the tumor volume was calculated according to the formula: V = D * d^2^/2. The growth inhibitory rate (IR) was calculated by the formula: IR(%) = 100 ‒ [(V1/V2)] * 100, wherein V1 is the mean tumor volume in the mAb treated group, and V2 is the mean tumor volume in the control group. Animals bearing tumors with D ≥ 15 mm or with signs of ulceration were sacrificed for humane reasons.

The *in vivo* experiments in the subcutaneous model continued until two mice in a group developed tumor size with D ≥ 15 mm. This was observed on day 27 in the control group. Since the ethical protocol requires that these two mice should be sacrificed we decided to sacrifice all the mice to perform a comparative study.

Mice were euthanized and the tumors and organs (spleen, bone marrow, brain, lungs, liver, small intestine, ovaries and spine) were harvested and weighed. Organs were fixed in 4% neutral buffered formaldehyde for histochemistry analysis whereas a piece of fresh spleen and the bone marrow were conserved in PBS at 4°C for flow cytometry assays. Subcutaneous tumors were divided in two: one half was fixed in 4% neutral buffered formaldehyde for histochemistry analysis and the other half was conserved in PBS at 4°C for flow cytometry assays. In the intravenous model, mice were euthanized when they developed incipient signs of limb paralysis, approximately 6–9 weeks after the inoculation of the lymphoma cells.

In order to evaluate potential toxic effects of the anti-human CCR7 mAb, a third group of 3 mice were not inoculated with tumor cells but treated with the anti-human CCR7 mAb following the same administration regimen than that of the treated xenografted mice.

### Flow cytometric cell analysis

Spleens and tumors were mechanically disaggregated. Cells were harvested and washed twice in cold PBS. Red cells were lysed using ammonium chloride solution (BD Biosciences), and then the remaining cells were washed twice with cold PBS, resuspended in binding buffer (BD Biosciences), and counted. One million cells from the spleen, bone marrow or tumors were incubated with PB anti-human CD20 mAb (clone 2H7, specific for human B cells, non cross reacting with murine lymphoid cells) in 50 μl of blocking solution (PBS, 2% BSA) for 15 minutes. The appropriate isotype control was included in the analysis. Analysis was performed on a FACSCanto II flow cytometer using the DIVA software (BD Biosciences).

### Apoptosis assay (viability assays)

The Annexin-V-FITC assay (BD Biosciences) was used according to the manufacturer’s instructions to quantitatively determine the percentage of non-viable cells following exposure to anti-human CCR7 mAb. Briefly, tumor cells were harvested and resuspended in binding buffer (BD Biosciences). 100 μl of the cell suspension was stained with Annexin-V-FITC. After 10 minutes, 0.4 ml of binding buffer and 10 μl of 7-AAD were added, and the cells were analyzed immediately by flow cytometry. Early apoptotic cells were defined as Annexin-V^+^/7-AAD^-^, late apoptotic cells as Annexin-V^+^/7-AAD^+^, dead cells as Annexin-V^-^/7-AAD^+^ and viable cells as Annexin-V^-^/7-AAD^-^.

### Immunohistochemistry

Organs and tissues extracted from mice were fixed in 4% formalin, dehydrated through grades of ethanol in a Microm STP 420D sample processor (Thermo Scientific, Kaklamazoo MI, USA), embedded in paraffin in EC 350–1 paraffin station (Myr, El Vendrell, Tarragona, Spain) and sliced in 4 μm sections. For human CD20 inmmunohistochemistry, the antigen retrieval was performed with citrate pH 6 in microwave. Slides were stained with anti-human CD20 antibody (clone L26, Roche) by using the VENTANA BenchMark ULTRA System (Roche).

### Statistical analysis

Statistical significance between untreated and treated samples was assessed with Student’s t test for unpaired data (two tail). Differences were considered significant if P-values were < 0.05. Survival data were analyzed by the Kaplan-Meier method and the Tarone-Ware test was used to test for significance among all the groups.

## Results

### Anti-CCR7 mAb blocks migration of mantle cell lymphoma cells in response to CCL19 and CCL21 in vitro and mediates CDC and ADCC

CCR7 is expressed in different primary lymphoproliferative disorders as we and others have previously demonstrated (12–18). In this study, we have confirmed and extended these results in a larger cohort of patients. Our results show that tumor cells from CLL and MCL patients consistently express CCR7 on the cell surface and at high density, compared to other lymphoproliferative syndromes. Other lymphomas such as follicular lymphomas or lymphoplasmacytic lymphomas also contain tumor populations expressing CCR7 but mixed with CCR7-negative tumor cells (see Additional file 1: Figure S1). Therefore, CCR7 may represent a new therapeutic target for the treatment of certain hematological cancers, in particular MCL and CLL.

**Figure 1:**
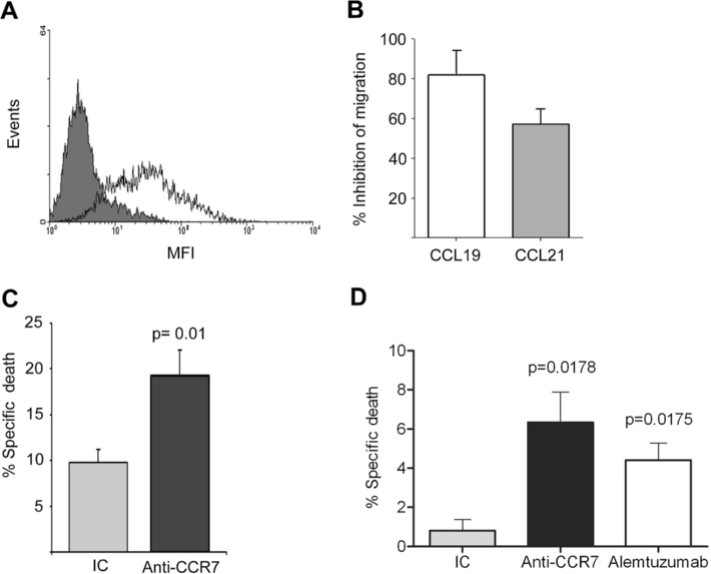
**In vitro mechanisms of action of anti-CCR7 mAb. (A)** Expression of CCR7 on MCL cell line Granta-519 by flow cytometry. Cells were co-stained with anti-CD19 monoclonal antibody (mAb) and either isotype control mAb (grey area) or anti-human CCR7 mAb (white area). CCR7 surface density was measured as mean fluorescence intensity (MFI). One representative experiment is shown. **(B)** Anti-CCR7 mAb abrogated the migration of Granta-519 cells. Cells were preincubated with anti-CCR7 mAb (2 μg/ml) or the respective irrelevant isotype control and then exposed to CCL19 or CCL21 for 4 hours in a transwell assay. **(C)** Anti-CCR7 mAb mediates specific CDC in Granta-519 cells. Cells were incubated with anti-CCR7 mAb (2 μg/ml) or the respective irrelevant isotype control (IC) and then exposed to rabbit complement for 1 h. Cell lysis was determined by 7-AAD incorporation in flow cytometry. The fold induction of cell lysis as a result of CDC is shown here. Bars represent mean +/- SD of six experiments. P-value refers to the difference of cell lysis fold induction mediated by an IC or an anti-CCR7 mAb. **(D)** Anti-CCR7 mAb mediates ADCC of Granta-519 cells. Calcein-UV-labeled Granta-519 cells previously incubated either with isotype control (IC), anti-CCR7 mAb (100 μg/ml) or alemtuzumab (100 μg/ml), were plated with human PBMC at an effector to target ratio of 10:1 for 24 hours. The average percentage of specific lysis +/- SEM from three independent experiments is shown. P-values refer to the differences of specific lysis mediated by anti-CCR7 mAb or alemtuzumab and the IC.

To assess the ability of anti-CCR7 antibodies to inhibit *in vitro* migration of MCL cells towards the ligands of CCR7, the homeostatic chemokines CCL19 and CCL21, we used the CCR7-positive Granta-519 MCL cell line (Figure 1A). As shown in Figure 1B, we demonstrated in transwell chamber assays that anti-CCR7 mAb (clone 150503) was very efficient in preventing Granta-519 cells chemotactic response to CCL19 or CCL21. Another anti-CCR7 mAb (clone2H4, IgM) was also able to partially block Granta-519 migration, albeit less effectively than clone 150503 (data not shown).

The ability of the anti-human CCR7 antibodies to induce CDC or ADCC in MCL cells was also evaluated in the MCL cell line Granta-519. To perform the CDC experiments, the cells were preincubated with two different clones of the anti-CCR7 antibody or with the corresponding isotype control (IC) for 1 hour and incubated with rabbit complement during an additional hour. The anti-CCR7 clone 150503 induced a two-fold induction in CDC when compared with the control antibody (Figure 1C). The ADCC experiments were performed with human PBMC and Granta-519 cells as targets in the presence of either IC antibody, the anti-CCR7 mAb or alemtuzumab, a therapeutic antibody known to mediate ADCC. We confirmed that human PBMC mediated significant cellular cytotoxicity through the engagement of either anti-CCR7 (p = 0.0178) or alemtuzumab (p = 0.0175) (Figure 1D).

### Anti-CCR7 mAb delays the appearance of tumors in an early-treated subcutaneous model of mantle cell lymphoma

These *in vitro* results described above prompted us to question whether the anti-CCR7 mAbs might also block MCL cells migration towards the anatomic sites producing CCL19 and CCL21 and trigger MCL cells cell death *in vivo*. To assess the *in vivo* effects of anti-CCR7 mAb, Granta-519 MCL cells were xenografted in NOD/SCID mice. Two models were studied: cells were inoculated either subcutaneously or intravenously in the tail vein, to produce either localized tumors or to generate diffuse lymphoma in the mice over time. Also, the 150503 anti-CCR7 mAb clone was used for these *in vivo* experiments in virtue of its effectiveness inhibiting the *in vitro* migration of MCL cells and mediating CDC.

**Figure 2:**
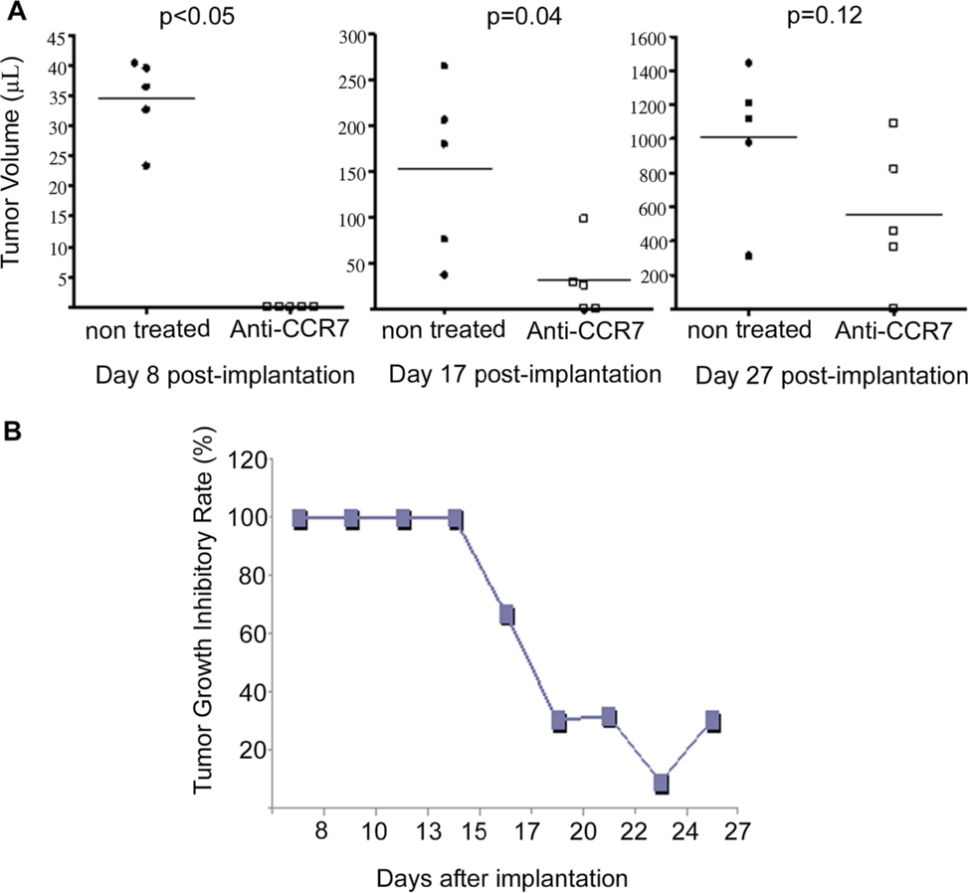
**Anti-CCR7 mAb reduces tumor growth of MCL cells.** Granta-519 cells were implanted subcutaneously in the right flank of NOD/SCID mice (n = 10). Treatment started at day 2 post-inoculation and continued at days 6 and 10. Anti-CCR7 mAb were administered intraperitoneally. Once subcutaneous tumors were palpable (≥4 mm) the diameters of the tumor mass was measured every 3 days. **(A)** Effect of anti-CCR7 mAb treatment on tumor volume in Granta-519 xenograft-bearing mice. An altered growth pattern in the treated and control groups are shown in the graph. The time points correspond to day 8 (first palpable tumors), day 17 and day 27. Each black dot (●, control group) or white square (□, treated group) represents one individual mouse. Horizontal bars represent mean tumors volume of each group, which consisted of 5 mice. Tumor volumes were calculated according to the V = D * d^2^/2 formula, as stated in Design and methods. P-values refer to the tumor volume differences from mice treated with PBS or anti-CCR7 mAb at different time points. **(B)** Anti-CCR7 mAb delays the development of subcutaneous tumors. The maximum growth inhibitory rate (IR) was reached within the first two weeks post anti-CCR7 mAb administration. The inhibitory rate is shown and is given by IR(%) = 100 ‒ [(V1/V2)] * 100 where V1 is the mean tumors volume in the mAb treated groups and V2 is the mean tumors volume in the control group.

The subcutaneous model of MCL was generated by subcutaneous implantation of 5 × 10^6^ Granta-519 cells in the right flank of NOD/SCID mice. To determine the efficiency of the anti-CCR7 therapy in the early stages of tumor implantation we established two groups of mice: 1) the treated group (n = 5), that received intraperitoneally 200 μg of anti-CCR7 mAb on days 2, 6 and 10 after the Granta-519 cells inoculation, and 2) the control group (n = 5), that was treated with PBS following the same schedule than that of the treated group. The treatment was stopped after day 10 because a significant therapeutic effect was already observed. For comparative reasons, all animals were sacrificed at day 27 as described in materials and methods section. The first measurable subcutaneous tumors were detectable at day 8 in the control mice (Figure 2A, day 8). In contrast, the treatment with the anti-human CCR7 mAb significantly delayed the tumor appearance and the first subcutaneous tumors were observed at day 17 in three out of five mice (Figure 2A, day 17). Differences in tumor volume were also observed between the two groups by the end of the study (Figure 2A). Tumor growth inhibition was evident until day 17 in the mice treated with anti-CCR7 mAb (Figure 2B), even though treatment was stopped at day 10 post-inoculation. At the end-point of the study, the size of the tumors in the untreated group was clearly larger than that of the treated group (Figure 2A, day 27).

### Mechanism of the *in vivo* tumoricidal activity of the anti-human CCR7 mAb in the subcutaneous model

**Figure 3:**
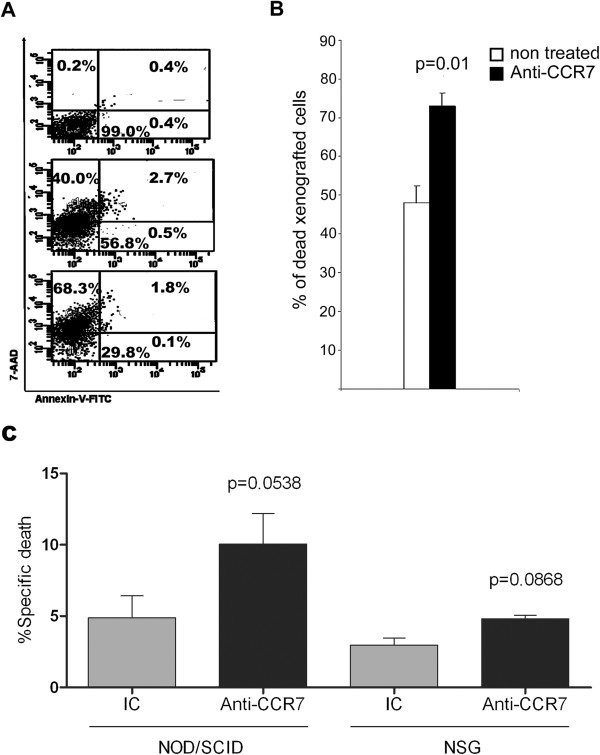
**Anti-human CCR7 mAb induces cell death in xenografted Granta-519 cells from the subcutaneous tumors.** The Annexin-V-FITC/7-AAD assay was used to quantitatively determine the percentage of non-viable cells following exposure to the anti-CCR7 mAb. **(A)** Granta-519 cells maintained in culture medium supplemented with 10% FCS (upper dot plot). Xenografted Granta-519 cells harvested from subcutaneous tumors in the control group (middle dot plot). Xenografted Granta-519 cells recovered from subcutaneous tumors in the CCR7 mAb-treated group (bottom dot plot). The Annexin V/7-AAD double staining made it possible to distinguish among viable cells (Annexin-V-/7-AAD-), early apoptotic cells (Annexin-V+/7-AAD-), late apoptotic cells (Annexin-V+/7-AAD+) and dead cells (Annexin-V-/7-AAD+). A representative experiment is shown (n = 5). **(B)** Percentage of dead cells (Annexin-V-/7-AAD+) in tumors from the control group (white bar, n = 5) or the treated group (black bar, n = 5). The mean ± SEM is shown. P-value denotes significant differences between the percentage of dead cells in the subcutaneous tumors from PBS- and anti-CCR7 mAb-treated mice. **(C)** Splenocytes from NOD/SCID mice but not from NSG mice mediate anti-CCR7 mAb-dependent cellular cytotoxicity. Calcein-UV-labeled Granta-519 target cells previously incubated either with IC or anti-CCR7 mAb (100 μg/ml) were plated with mice splenocytes at effector to target ratio of 10:1 for 24 hours. The mean percentage of specific lysis +/- SEM is shown from seven independent experiments with NOD/SCID mice and three independent experiments with NSG mice. P-values refer to the differences between the specific death mediated by the IC and the anti-CCR7 mAb in the corresponding strain of mice.

The delay in the tumor growth exerted by the anti-human CCR7 mAb might involve the death of tumor cells by cytotoxicity. To verify this hypothesis, Granta-519 MCL cells were harvested from subcutaneous tumors at the end of the experiment (day 27) and were double stained with Annexin-V/7-AAD to assess cell viability (Figure 3A). Interestingly, we observed a significant increase in the percentage of non-viable cells in the CCR7 mAb treated group when compared to the control group (Figure 3B). This result supports the notion that the anti-CCR7 mAb is able to induce *in vivo* cytotoxicity probably mediated by NK cells as the NOD/SCID mice lack functional complement and cytotoxic T cells. Indeed, a significant ADCC activity was mediated by splenocytes from NOD/SCID mice through the engagement of the anti-CCR7 mAb causing Granta-519 MCL cell death (Figure 3C). Confirming that ADCC was mediated by NK cells, splenocytes from NSG mice, which are completely devoid of NK and cytotoxic T cells, did not induced significant ADCC in Granta-519 cells (Figure 3C).

### Anti-human CCR7 mAb reduces dissemination of tumor cells in distant organs in the subcutaneous model

**Figure 4:**
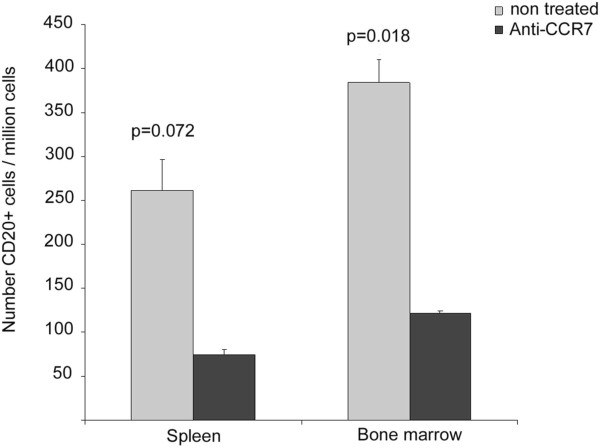
**Anti-human CCR7 mAb reduces tumor cell infiltration in bone marrow and spleen in the subcutaneous model of the MCL.** One million cells from spleen and bone marrow were harvested and incubated with anti-human-CD20 mAb and the respective irrelevant isotype control. Then, the number of xenografted Granta-519 cells migrated to bone marrow and spleen in control mice (grey bar) and treated mice (black bar) was quantified. The mean ± SE is shown. P-values refer to the differences of infiltration in tissues from PBS- and anti-CCR7 mAb-treated mice.

The extent of tumor dissemination was assessed by flow cytometry analysis of cell suspensions obtained from spleen and bone marrow at 27 days after subcutanenous implantation (Figure 4). Lymph nodes were nearly undetectable due to the immunodeficient status of the NOD/SCID mice and the relatively short follow up of the model. Interestingly enough, there was a significant reduction (p = 0.018) in the number of the infiltrating Granta-519 MCL (human CD20+) cells in the bone marrow samples from the treated group (122 ± 25.5 per million of total cells) compared to the number of infiltrating tumor cells in the control group (384 ± 64.4 per million of total cells). Infiltrating human CD20+ cells were also reduced in the spleen of treated group (74 ± 35.6 per million of total cells) compared to the control group (261 ± 72.5 per million of total cells), although it did not reach statistical significance (p = 0.072). No evidence of metastases in non-lymphoid organs was found in either group of mice, which could be explained by the lack of time for the cells to migrate into these other organs (data not shown).

### Anti-CCR7 mAb prevents tumor growth in peri-implantation and post-implantation Granta-519 MCL xenogratf models

**Figure 5:**
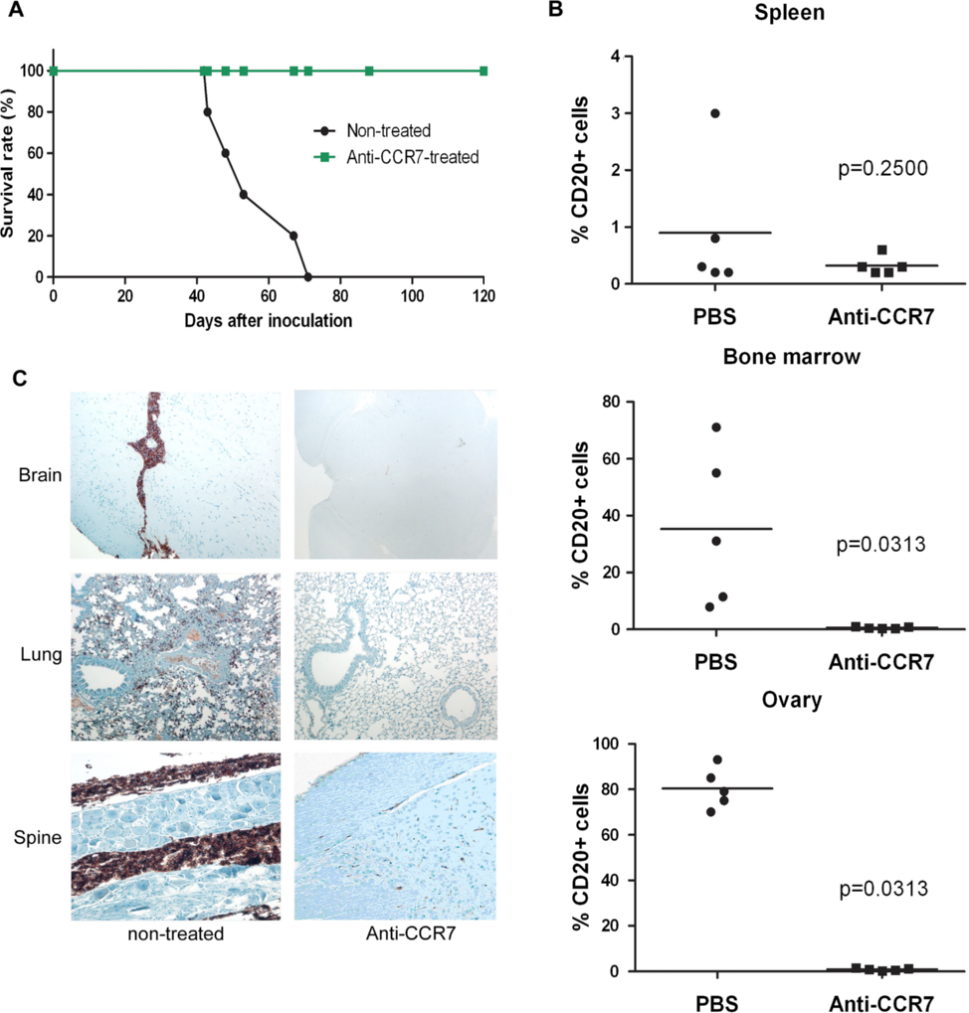
**Survival and organ infiltration in the peri-implantation model of disseminated MCL. (A)** Anti-human CCR7 mAb significantly increases the survival of xenografted mice**.** Kaplan-Meier estimates of overall survival of mice treated with the anti-CCR7 mAb. Granta-519 cells were implanted intravenously in the vein tail of NOD/SCID mice (n = 10). Treatment with 200 μg of the anti-CCR7 mAb (■) or with PBS (●) started at day 2 post-inoculation and continued at days 6 and 10. **(B)** and **(C)** Anti-human CCR7 mAb significantly reduces the infiltration of lymphoid and distant organs. **(B)** One million cells from spleen, bone marrow and ovaries were harvested and incubated with anti-human-CD20 and the respective irrelevant isotype control. Then the percentage of xenografted CD20+ Granta-519 cells migrated to bone marrow, spleen and ovaries are shown. P-values refer to the differences of infiltration in tissues from PBS- and anti-CCR7 mAb-treated mice. Each black dot (●, control group) or black square (■, treated group) represents one individual mouse. Horizontal bars represent mean percentage of xenografted CD20+ Granta-519 cells of each group, which consisted of 5 mice. **(C)** Immunohistochemistry assays with anti-human CD20 mAb were performed in tissue sections from brain, spine and lungs. Representative pictures from both a control mouse and an anti-CCR7- treated mouse are shown.

The intravenous model of MCL with Granta-519 cells is characterized by infiltration of different lymphoid organs, mostly bone marrow, and of the CNS, in particular lumbar spine nerves infiltration, causing hind leg paralysis of the xenografted mice. The mice in the control group were all sacrificed between days 42 and 71 when the first signs of hind leg paralysis were evident, with a median survival time of 56 days. Remarkably, all mice treated with anti-CCR7 mAb starting 2 days after inoculation (peri-implantation model) remained alive at the time when the last mouse in the control group had to be euthanized. These mice treated with the anti-CCR7 mAb did not develop any clinical sign and survived up to 120 days of observation which could be considered a *bona fide* disease-free period (Figure 5A). At the time of the sacrifice, between days 42 and 71 after inoculation in the untreated group and on day 120 in the treated group, several tissues and cells from different lymphoid organs were collected to study the degree of lymphoma development and infiltration. In untreated mice the percentage of positive CD20+ Granta-519 cells in bone marrow ranged from 8% to 71% whereas in spleen was nearly undetectable (Figure 5B). Lymph nodes were only found in two out of five control mice, and these nodes were mainly populated by human CD20+ cells (data not shown). Conversely, there was a consistent infiltration of certain non-lymphoid organs such as the ovaries (Figure 5B), spinal cord, brain and lungs (Figure 5C). In contrast, and consistent with the survival data, we could not find any Granta-519 cells in the different organs from mice treated with the anti-CCR7 mAb (Figure 5B and C).

**Figure 6:**
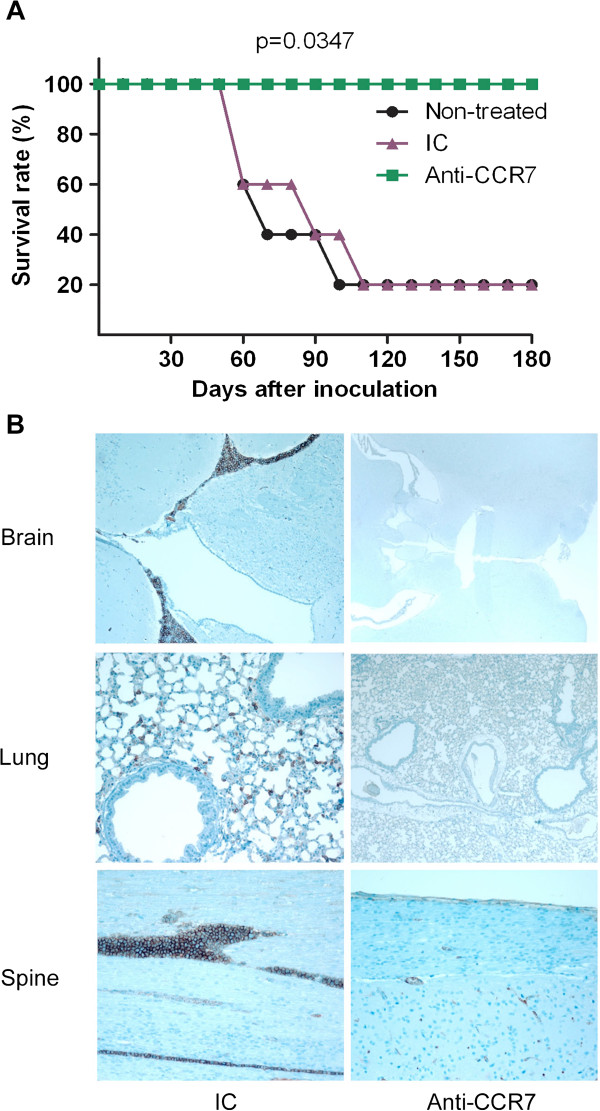
**Survival and organ infiltration in the post-implantation model of disseminated MCL. (A)** Anti-human CCR7 mAb significantly increases the survival of xenografted mice. Kaplan-Meier analysis of overall survival of mice treated with the anti-CCR7 mAb. Granta-519 cells were implanted intravenously in the vein tail of NOD/SCID mice (n = 15). Treatment with 200 μg of either the anti-CCR7 mAb or its corresponding isotype control or with PBS started at day 7 post-inoculation and continued at days 11 and 15. Anti-CCR7 mAb were administered intraperitoneally. P-value refers to the differences of the survival of PBS-, IC- and anti-CCR7-treated groups **(B)** Anti-human CCR7 mAb significantly reduces the infiltration of distant organs in the post-implantation model. Immunohistochemistry analyses were performed in tissue sections from brain, spine and lungs using anti-human CD20 mAb. Representative pictures from both a control mouse and an anti-CCR7 treated mouse are shown.

Due to the high therapeutic efficacy of the anti-CCR7 mAb described in this section, the potential of the antibody was further studied in a post-implantation disease model in which surviving Granta-519 MCL cells might have already migrated to their target organs and be less exposed to the anti-CCR7 treatment, thus better representing a clinical scenario in humans. For this purpose, the treatment was initiated seven days after the intravenous inoculation of the lymphoma cells. In these new set of experiments, three groups of five mice each were treated with 200 μg of the anti-CCR7 mAb, 200 μg of its corresponding IC or with PBS (the vehicle of administration), that were administered intraperitoneally on days 7, 11 and 15. The survival rate in both PBS and IC groups was 20% with an average survival of 68 days in the PBS group and 75 days in the IC group. Remarkably, 100% of the mice treated with anti-CCR7 were alive after 6 months (Figure 6A). At the time of the sacrifice, between days 56 and 109 after inoculation in the IC group and on day 180 in the treated group, several tissues were collected and analyzed. Similar to the peri-implantation disease model there was a constant infiltration of the CNS and other distant organs including lungs, in both the PBS and the IC groups, which was prevented by the anti-CCR7 mAb (Figure 6B).

## Discussion

In this study, we provide preclinical proof of concept of the efficacy of the anti-CCR7 therapy in a MCL model. CCR7 mediates the classical chemotaxis and extravasation pathways of lymphoid cells into the targeted organs but also it participates in cell invasion by promoting extracellular matrix degradation through the secretion of metalloproteases [28]. In addition, the ligands of CCR7 have been demonstrated to mediate prosurvival signals in both epithelial and lymphoid neoplasias [20,29-31]. In summary, the chemokine receptor CCR7 enables lymphoma cells to enter and disseminate in anatomic niches where they received different pro-survival signals including those from CCR7 ligands themselves [3]. For all these reasons, therapeutic effects of anti-CCR7 mAbs would not be restricted to classic complement or cellular-dependent cytotoxicity, but also would imply the blockage of migration and invasive signals derived from the activated CCR7, thus preventing lymphoma cells migration to lymphoma supporting-niches.

Consistent with a critical role of CCR7-regulated cell migration in lymphoma development, it has been described that CCR7–deficient lymphoma cells have a significant delay in the onset of lymphoma compared to CCR7+ lymphoma cells [32]. Indeed, it was demonstrated that such delay in lymphoma progression was related to the blockage of migratory signals evoked by CCR7 since the absence of this chemokine receptor did not alter the proliferative and/or apoptotic rates of the CCR7-/- lymphoma cells.

The well-known role of tumor microenvironment as a driver of tumor survival and promoter of drug resistances turns it as a suitable target for pharmacology development aimed to block stroma’s survival signals driving the progression of the disease. Among those targets are CXCR4 antagonists [33], PI3K, Btk or Syk inhibitors [34,35], antibodies directed against CD44 [36] or combined therapies [37].

A combined anti-CCR7 therapy might include an anti-CCR7 mAb and inhibitors of the signaling pathways activated by the CCR7 ligands, including inhibitors of the kinases PI3K, ROCK and ERK which efficiently block both CCR7-mediated migration and survival *ex vivo* in primary CLL cells from patients [20]. In the particular case of the MCL, it is known that BCR-associated kinases Syk and Btk are the most abundantly expressed phosphoproteins [38], suggesting that BCR signaling plays a central role in disease maintenance and progression. The inhibitors of Syk and Btk have already entered the clinical stage [22]. It would be very interesting to investigate whether CCR7 blockage with anti-CCR7 mAbs would cooperate with these kinases inhibitors to abrogate MCL growth and survival.

In our experiments mice were administered with 200 μg per inoculation (30 mg/m^2^). Considering that rituximab is given to patients at a dose of 375 mg/m^2^, a therapy with anti-CCR7 seems feasible in human patients. Mice treated with this dosage of anti-CCR7 mAb did not have any evident unwanted effect caused by the treatment (with or without the inoculation of the lymphoma cells) even six months after the administration of the anti-CCR7 mAb (data not shown). However, these mice were immunodeficient, and further research is required to assess whether an anti-CCR7 therapy could result in any kind of immunodeficiency, since the anti-CCR7 antibody could both eliminate normal naïve and central memory lymphocytes and inhibit their entry into SLO. However, if any immunodeficiency is triggered by the anti-CCR7 therapy, it should be expected that while it could be greater than that caused by the treatment with rituximab, which only eliminates B cells, it would be milder than that caused by alemtuzumab treatment, which eliminates all T and B lymphocytes as well as other leukocyte subpopulations [39,40].

On the other hand, an anti-CCR7 therapeutic strategy would also eliminate or abrogate the migration of other CCR7-expressing cell populations like natural regulatory T cells or the semi-mature dendritic cells, which contribute to the induction and maintenance of the tolerance [41,42]. In this regard, it has been published that the chronic absence of CCR7 may lead to the development of autoimmune diseases given the role of CCR7 in the maintenance of the tolerance [43]. However, targeting the CCR7 molecule on T cells during the limited period of the therapeutic window may also overcome tumor tolerance mediated by CCR7+ regulatory T cells, in addition to both the tumoricidal effect exerted by the anti-CCR7 mAb and the abrogation of the nodular dissemination and metastasis of tumors expressing this chemokine receptor. Moreover, it is worth mentioning that blocking the entry of normal CCR7-positive T lymphocytes into SLO may be beneficial because once inside the SLO they support the growth of B cells either normal or tumoral, through certain costimulatory molecules such as CD40L, which activates CD40 on the surface of B cells [44].

The beneficial effects of an anti-CCR7 therapy would not be limited to the blocking of the migration of CCR7-expressing cells towards SLO but also towards different anatomic sites including CNS which represents one of the most important sanctuaries of the hematological malignancies. For this reason, it would be very interesting to identify those malignant hemopathies in which CCR7 expression correlates with CNS infiltration, as it is the case for T-cell acute lymphoblastic leukemia [18]. It is worth noting, however, that CLL, which is the chronic lymphoproliferative disorder having the highest expression levels of CCR7, does not usually infiltrate neural tissues. This suggests that other factors besides CCR7, such as adhesion molecules and metalloproteases might be involved in the colonization of CNS.

Apart from its role in the dissemination of hematologic malignancies, CCR7 is responsible, at least in part, for the ganglionar dissemination of solid epithelial tumors including breast, colorectal, head and neck and gastric carcinomas [45] as well as melanoma [46]. Unpublished results from our group indicate that the activation of CCR7 in the breast cancer cell line MCF7 leads to the activation of the oncoprotein cortactin and the promotion of cellular structures essential for metastasis like lamellipodia and invadopodia. Interestingly, the treatment with a blocking anti-CCR7 mAb abrogated both lamellipodia and invadopodia formation, further supporting the role of CCR7-mediated signaling in cancer progression.

In our current study, the mice with the subcutaneous lymphoma that were treated with the anti-CCR7 mAb developed substantially less metastasis to distant organs when compared with the untreated mice suggesting a potential therapeutic role to prevent metastasis in those primary tumors expressing this chemokine receptor. Further studies to demonstrate an antimetastatic effect of the anti-CCR7 therapy in an ortothopic model of breast carcinoma with MCF7 cells are warranted.

In conclusion, the data presented here demonstrate that the anti-CCR7 mAb has a notable anti-tumor efficacy, causing a significant delay of the tumor growth rate and metastatic process in the subcutaneous model and also hindering lymphoma cells dissemination in the intravenous model. Our results support that anti-CCR7 therapy might be indicated for patients suffering of CCR7-positive B cell non-Hodgkin lymphoma and CLL. Our study open a way for the development of different therapeutic protocols in which the current chemotherapy could be used in combination with antagonists of either CCR7 expression or function, including anti-CCR7 mAbs and pharmacologyc inhibitors of CCR7-signaling pathways.

## Competing interests

MAP, CCM, JPV and FT are employees of IMMED, the company which is maintaining the patent that protects the use of anti-CCR7 antibodies to treat cancer.

## Authors’ contributions

CMC was the principal investigator, conceived the study and takes primary responsibility for the paper. BSC, MAP and CCM performed laboratory work, data collection and analysis. CC, ABN and GPC contributed to laboratory work and review of the manuscript. CGA performed and analyzed the immunohistochemistry experiments. FT, JPV and EFR contributed to research design and to the review of the manuscript. CMC and JMZ coordinated the research. CMC and JMZ wrote the paper. All authors read and approved the final manuscript.

## Supplementary Material

Additional file 1: Figure S1 Expression of CCR7 in different B cell malignancies. Surface CCR7 expression of different B cell neoplasms was analyzed by flow cytometry and expressed as percentage of CCR7+ cells (A) or as the MFI of CCR7-positive cells (B). Normal B cells (n = 4); B-cell acute lymphoblastic leukemia (B-ALL) (n = 3); Mantle cell lymphoma (MCL) (n = 6); Follicular lymphoma (FL) (n = 9); Splenic marginal zone lymphoma (SMZL) (n = 3); Hairy cell leukemia (HCL) (n = 4); Lymphoplasmacytic lymphoma (LPL) (n = 9); Multiple myeloma (MM) (n = 10); atypical CD5- B-cell chronic lymphocytic leukemia (CD5- B-CLL) (n = 5); typical CD5+ B-cell chronic lymphocytic leukemia (CD5+ B-CLL) (n = 79). Click here for file
