# Supplementary figures and images for: Anti-CCR7 therapy exerts a potent anti-tumor activity in a xenograft model of human mantle cell lymphoma
Source: J Hematol Oncol. 2013 Dec 4;6:89. doi: 10.1186/1756-8722-6-89 (PMC3879031; doi:10.1186/1756-8722-6-89)

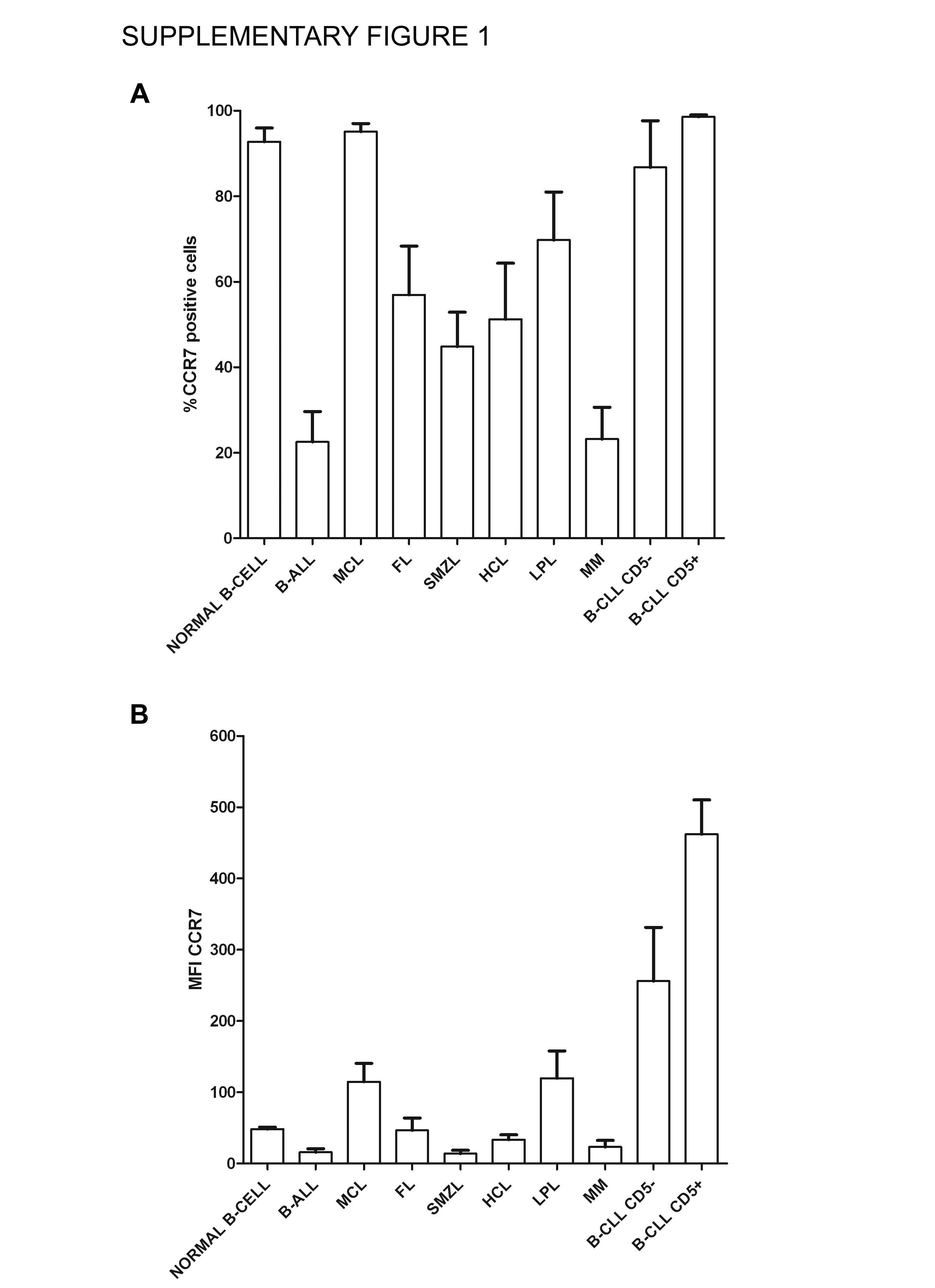

Supplement: Additional file 1: Figure S1 — Expression of CCR7 in different B cell malignancies. Surface CCR7 expression of different B cell neoplasms was analyzed by flow cytometry and expressed as percentage of CCR7+ cells (A) or as the MFI of CCR7-positive cells (B). Normal B cells (n = 4); B-cell acute lymphoblastic leukemia (B-ALL) (n = 3); Mantle cell lymphoma (MCL) (n = 6); Follicular lymphoma (FL) (n = 9); Splenic marginal zone lymphoma (SMZL) (n = 3); Hairy cell leukemia (HCL) (n = 4); Lymphoplasmacytic lymphoma (LPL) (n = 9); Multiple myeloma (MM) (n = 10); atypical CD5- B-cell chronic lymphocytic leukemia (CD5- B-CLL) (n = 5); typical CD5+ B-cell chronic lymphocytic leukemia (CD5+ B-CLL) (n = 79). [file 1756-8722-6-89-S1.jpeg]
